# The gills and skin microbiota of five pelagic fish species from the Atlantic Ocean

**DOI:** 10.1007/s10123-024-00524-8

**Published:** 2024-05-13

**Authors:** José Luis Varela, Eleni Nikouli, Antonio Medina, Sokratis Papaspyrou, Konstantinos Kormas

**Affiliations:** 1 Department of Biology, University of Cádiz, Puerto Real, 11510 Cádiz, Spain; 2 Department of Ichthyology and Aquatic Environment, School of Agricultural Sciences, University of Thessaly, 384 46 Volos, Greece; 3 Agricultural Development Institute, University Research and Innovation Centre “IASON”, Argonafton & Filellinon, 382 21 Volos, Greece

**Keywords:** Microbiome, Host-microbe interactions, Fisheries

## Abstract

The gills and skin microbiota and microbiome of wild fish remain far more under-investigated compared to that of farmed fish species, despite that these animal-microbe interactions hold the same ecophysiological roles in both cases. In this study, the gills and skin bacterial microbiota profiles and their presumptive bacterial metabolisms were investigated in five open-sea fishes: bullet tuna (*Auxis* sp.), common dolphinfish (*Coryphaena hippurus*), Atlantic little tunny (*Euthynnus alletteratus*), Atlantic bonito (*Sarda sarda*) and Atlantic white marlin (*Kajikia albida*). Gills and skin tissues were collected from two to three individuals per species, from specimens caught by recreational trolling during summer of 2019, and their bacterial 16S rRNA gene diversity was analysed by high-throughput sequencing. The gills bacterial communities among the five species were clearly different but not the skin bacterial microbiota. The dominant operational taxonomic units belonged to the Moraxellaceae, Pseudomonadaceae, Rhodobacteraceae, Staphylococcaceae and Vibrionaceae families. Despite the differences in taxonomic composition, the presumptive bacterial metabolisms between the gills and skin of the five fishes investigated here were ≥ 94% similar and were dominated by basic metabolism, most likely reflecting the continuous exposure of these tissues in the surrounding seawater.

**Supplementary Information:**

The online version contains supplementary material available at 10.1007/s10123-024-00524-8.

## Introduction

The microbial topography of fish microbiomes still remains biased towards the gastrointestinal and/or faecal material (Tarnecki et al. 2017; Egerton et al. 2018), apparently due to their importance in nutrition of farmed species, leaving the other two major microbial body sites, i.e. the gills and skin, under-investigated, especially for wild fish (Legrand et al. 2020; Diwan et al. 2023). Most of the available microbiome studies related to fish gills and skin microbiota involve some kind of disease state and, in most cases, refer to fish growing under commercial aquaculture or experimental (controlled) conditions (Diwan et al. 2023). Considering the high number of wild fish species and the importance of fish biomass in the ocean’s animal biomass (Bar-On et al. 2018), the taxonomic and functional microbiome diversity of the various fish tissue/organs remains practically untapped. This could be attributed to the multiple and ample challenges in wildlife microbiome studies, for which the frequently considered descriptive and exploratory work of microbiota profiling is considered the first major advancement and the first hypothesis-generating step towards the understanding of host-microbiome ecology in wild animal species, especially in body sites other than the gastrointestinal tract (Couch et al. 2022), such as the skin and gills in fish.

The skin microbiome of humans starts developing immediately after birth and is mostly influenced by factors related to the mother and nutrition/disease-associated ones, hygiene practices, co-housing with other individuals and/or animal/pets and environmental exposure (Santiago-Rodriguez et al. 2023). However, for fish, the factors affecting the microbiome development is not clear, since it is not known if skin microbiota are seeded from the egg and to what degree it is shaped by the surrounding microbial sea/water (e.g. (Nikouli et al. 2019)). Gills and skin are the tissues in immediate and constant exposure to the surrounding water’s microbiota, contrasting the other microbial hotspot of fishes, i.e. the gut habitat. Moreover, gills are considered the first organ that connects the fish with their external environment as they play a vital role in the gas exchange and defence, while both tissues are key players in processes such as osmoregulation, protection against abrasion, protection against environmental toxins and heavy metal toxicity, parental feeding, protection against pathogens and chemical communication (Reverter et al. 2018). Both of these mucosal surfaces, along with the microbes they bear, are considered key components for the fish’s well-being and health. In studies of fish whose health was compromised by microbial pathogens, it was shown that fish have evolved skin-related unique immune functions such as increased mucosal production for protection against microbial pathogens (Gómez & Balcázar 2008; Gomez et al. 2013; Merrifield & Rodiles 2015; Diwan et al. 2023). This leaves the ecological aspects and role of gills and skin microbiomes in healthy (especially wild) fish rather under-investigated.

Despite that the gills and skin microbiome are expected to have quite different physiological functions and be shaped by different factors than the gut microbiome (Legrand et al. 2020), an appropriate diet, one of the major factors responsible for maintaining healthy gut microbiomes, also contributes to gill-tissue integrity and functionality and enhances skin mucus production (Abdel-Latif et al. 2020; Firmino et al. 2020). Hence, the water-gills-skin-gut microbiomes of fish constitute a dynamic and interacting metamicrobiome (sensu (de Jonge et al. 2023)) (Troussellier et al. 2017). In this paper, we tested whether the structure and presumptive metabolic functions of the gills and skin bacterial microbiota are the same in five pelagic fishes, as these tissues are in constant contact with the surrounding water. We hypothesize that each of the five investigated fish species (a) selects for its specific gill and skin bacterial microbiota and (b) there are different and distinct gills and skin bacterial communities.

## Materials and methods

### Sample collection

Specimens of five pelagic fishes, bullet tuna (*Auxis* sp.), common dolphinfish (*Coryphaena hippurus*), Atlantic little tunny (*Euthynnus alletteratus*), Atlantic bonito (*Sarda sarda*) and Atlantic white marlin (*Kajikia albida*), were caught in the Gulf of Cadiz (East Atlantic Ocean) by recreational trolling during summer daylight hours in 2019. All the individuals were measured to the nearest 1-decimal cm (straight fork length or lower-jaw fork length) and weighed to the nearest 0.5 g (total weight) (Table S1). Small pieces of gill and skin were aseptically collected from each individual and stored at − 80 °C until analysis.

### Microbiota analysis and data processing

Bulk DNA was extracted from approximately 0.25 mg of gill and skin tissue with the QIAGEN QIAamp DNA Mini Kit (Qiagen, Hilden, Germany) according to the manufacturer’s protocol “DNA Purification from Tissues”. The extracted total DNA was used as a matrix to amplify the V3–V4 regions the bacterial 16S rRNA genes with the primer pair S-D-Bact-0341-b-S-17 and S-D-Bact-115 0785-a-A-21 (Klindworth et al. 2012). Sequencing of the amplified DNA fragments was performed on a MiSeq Illumina instrument (2 × 300 bp) at the MRDNA Ltd. (Shallowater, TX, USA) sequencing facilities. The raw DNA sequences from this study have been submitted to the Sequence Read Archive (https://www.ncbi.nlm.nih.gov/sra/) in the BioProject PRJNA1068742 (BioSample SAMN39602282). Processing of the raw 16S rRNA sequences was implemented with the MOTHUR software (v.1.45.3) according to its standard operating procedure (Schloss et al. 2009, 2011). Mitochondrial sequences were excluded, but sequences affiliated to phototrophic prokaryotes, namely Cyanobacteria and chloroplasts, were included in the rest of the analysis. The operational taxonomic units (OTUs) were determined at 97% cutoff similarity level and were classified with the SILVA database release 138 (Quast et al. 2013; Yilmaz et al. 2014). The final OTUs table was normalized to 20,497 and 15,768 reads per sample for the gills and skin bacterial microbiota, respectively. The Nucleotide Blast (http://blast.ncbi.nlm.nih.gov) tool was used for identifying the closest relatives of the resulting OTUs.

Non-metric multidimensional scaling (nMDS) based on the unweighted pair group method with the arithmetic mean Bray–Curtis similarity, and permutational multivariate analysis of variance (PERMANOVA) with 9999 permutations, was applied to detect differences between the gills and skin microbiota of the five fishes bacterial microbiota, which were performed using PAleontological STudies (PAST) software (Hammer et al. 2001). As is often the case with wild fish, the number of available individual samples to be analysed is not always reached, and for this, the number of individuals per fish species was rather low (Kormas et al. 2023). For this, we pooled individual samples from the same tissue of each fish species OTUs, to provide a more inclusive view of the overall bacterial microbiota per tissue and fish.

The presumptive metabolic functions of the gills and skin bacterial microbiota according to the 16S rRNA OTUs were made with the PICRUSt2 (Douglas et al. 2020) software. In order to obtain the gene family copy numbers of each OTU, a reference tree (with a NSTI cut-off value of 2) was built with the OTUs table. Functional annotation of the metabolic pathways was determined according to the Kyoto Encyclopedia of Genes and Genomes (KEGG) orthologs (Kanehisa & Goto 2000), Enzyme Classification numbers (EC), and Cluster of Orthologus genes (COGs) (Tatusov et al. 2000) and mapped according to the MetaCyc database (Caspi et al. 2019).

## Results

In gills, the mean number of OTUs varied between 110 (*S. sarda*) and 158 (*K. albida*) (Table 1). *S. sarda* showed the lowest diversity indices and *C hippurus* the highest. The OTUs richness in the skin varied between 82 (*S. sarda*) and 121 (*C. hippurus*), while the lowest diversity indices occurred in *K. albida* and highest in *S. sarda*. For all samples, the observed to predicted number of OTUs was ≥ 46% based on the Chao1 index. PERMANOVA showed that the gills bacterial microbiota among the five species were statistically clearly different (*p* < 0.001, *F* = 1.960) but not the skin microbiota (*p* = 0.056, *F* = 1.337).

**Table 1:** Alpha diversity metrics of the gills and skin bacterial microbiota of five pelagic fishes from the Atlantic Ocean (means ± SD). *OTUs* operational taxonomic units, *N* number of replicate samples

		*Auxis* sp. *N* = *3*	*Coryphaena hippurus* *N* = *2*	*Euthynnus alletteratus* *gills N* = *2, skin N* = *3*	*Sarda sarda* *N* = *3*	*Kajikia albida* *N* = *3*
No. of OTUs	Gills	134 ± 13	134 ± 27	115 ± 16	110 ± 19	158 ± 14
	Skin	105 ± 14	121 ± 23	97 ± 24	82 ± 14	116 ± 5
Simpson 1-D	Gills	0.91 ± 0.07	0.94 ± 0,01	0.88 ± 0.03	0.77 ± 0.22	0.88 ± 0.05
	Skin	0.91 ± 0.03	0.88 ± 0.10	0.83 ± 0.12	0.92 ± 0.02	0.78 ± 0.14
Shannon H	Gills	3.34 ± 0.58	3.62 ± 0.11	2.92 ± 0.19	2.76 ± 0.95	3.07 ± 0.48
	Skin	3.10 ± 0.25	3.14 ± 0.74	2.82 ± 0.51	3.10 ± 0.29	2.61 ± 0.57
Evenness J	Gills	0.24 ± 0.12	0.28 ± 0.03	0.16 ± 0.01	0.17 ± 0.10	0.15 ± 0.09
	Skin	0.22 ± 0.08	0.24 ± 0.19	0.18 ± 0.07	0.27 ± 0.04	0.13 ± 0.09
Coverage Chao1	Gills	0.460 ± 0.090	0.579 ± 0.016	0.621 ± 0.031	0.664 ± 0.124	0.641 ± 0.239
	Skin	0.662 ± 0.027	0.558 ± 0.009	0.750 ± 0.092	0.518 ± 0.159	0.573 ± 0.121

**Fig. 1:**
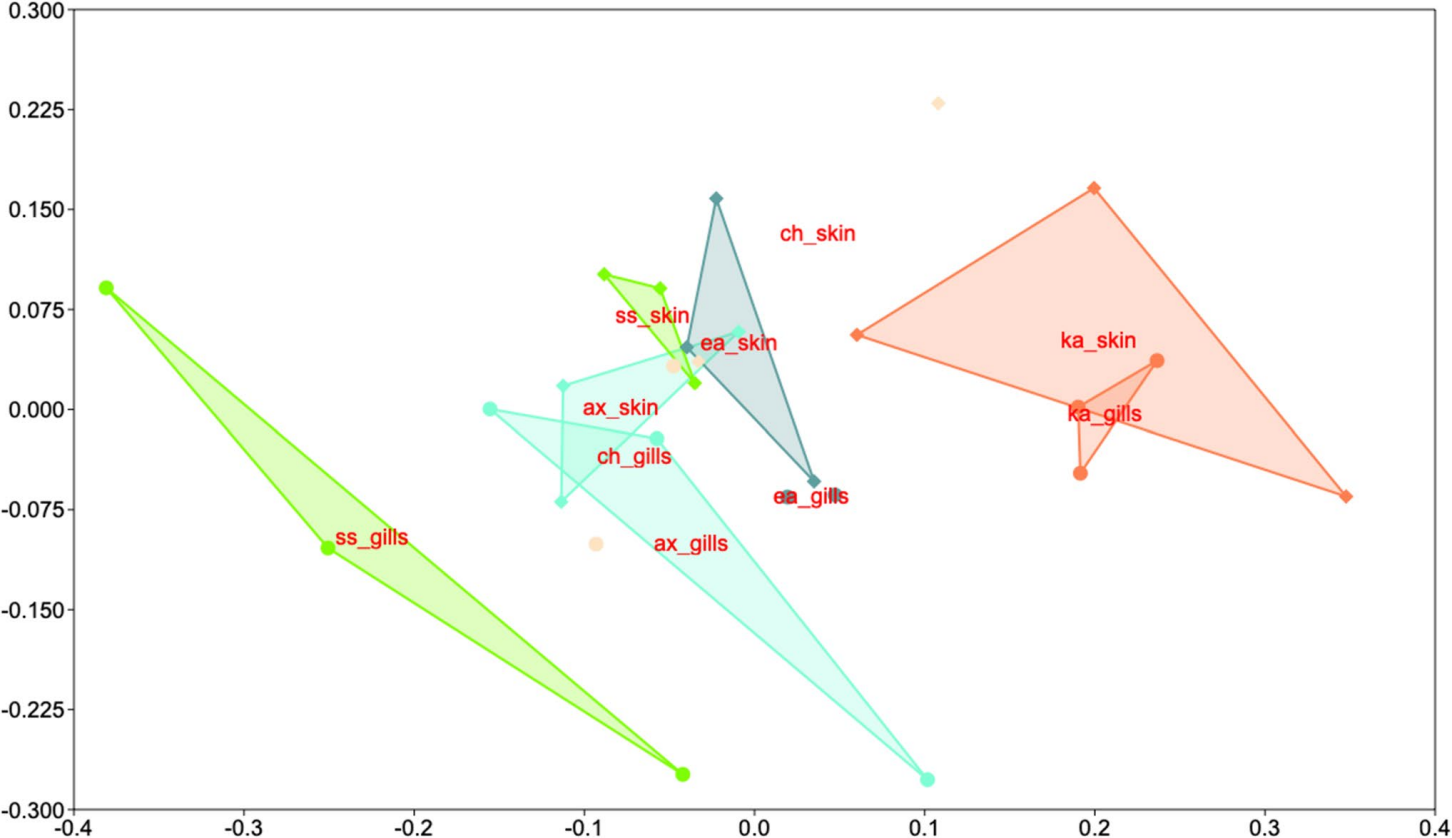
Non-metric multidimensional scaling of the gills (circles) and skin (diamonds) of five pelagic fishes from the Atlantic Ocean. Each colour refers to the same fish species: ax, *Auxis* sp.; ch, *Coryphaena hippurus*; ea, *Euthynnus alletteratus*; ss, *Sarda sarda*; ka, *Kajikia albida*

**Fig. 2:**
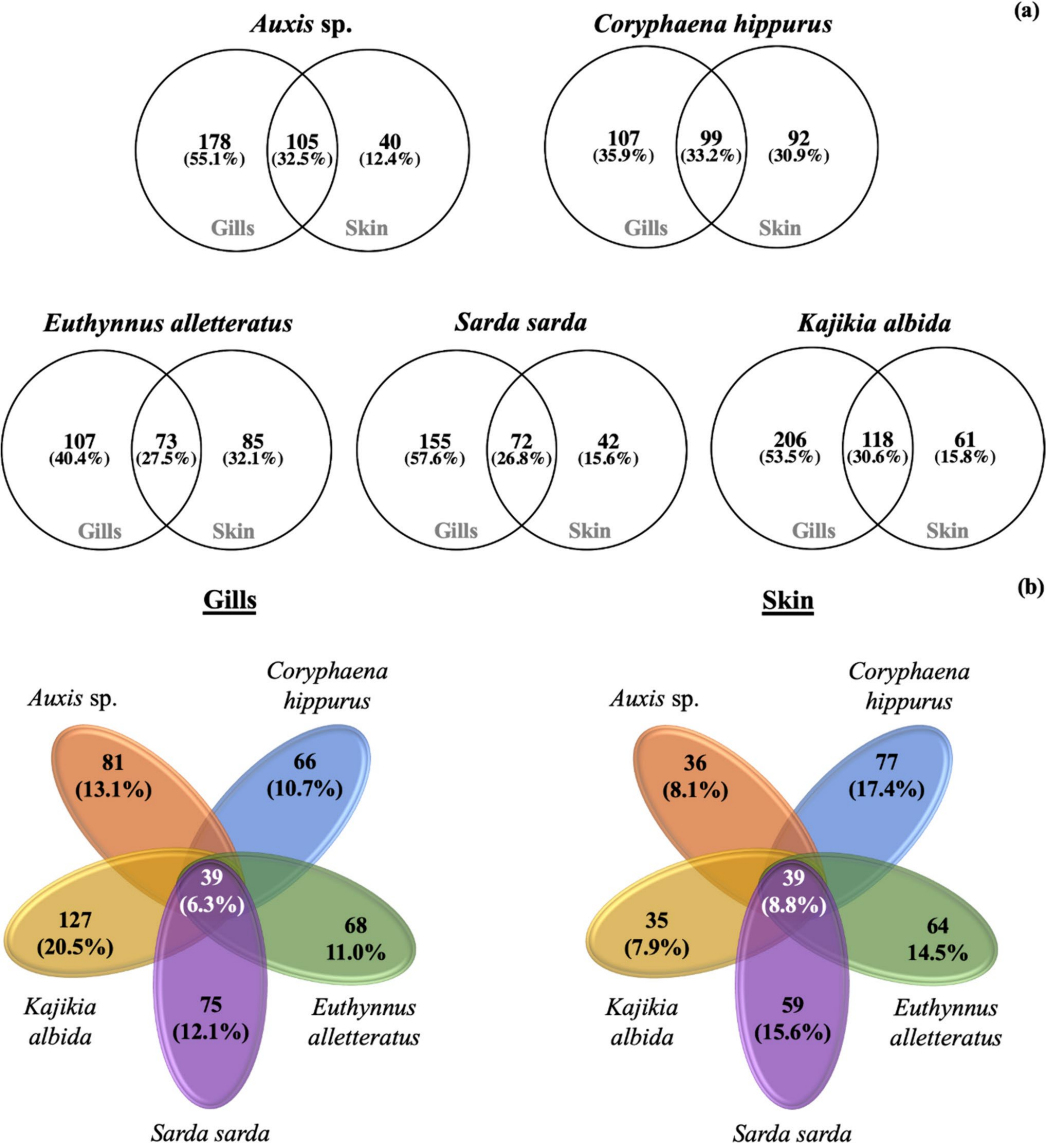
Overlap of the bacterial operational taxonomic unit richness between the gills and skin tissues (**a**) and in each of these tissues (**b**) of five pelagic fishes from the Atlantic Ocean

*S. sarda* showed the clearest distinct bacterial microbiota profile from its skin (Fig. 1). For all pairs of gills-skin bacterial OTUs, the gills had the highest number of unique OTUs (35.9% in *K. albida* to 57.6% in *S. sarda*), while the overlap percentage between the two tissues varied between 26.8 (*S. sarda*) and 33.2% (*C. hippurus*) (Fig. 2a). The five fishes shared 6.3% and 8.8% of their total OTUs richness for their gills and skin, respectively (Fig. 2b).

**Fig. 3:**
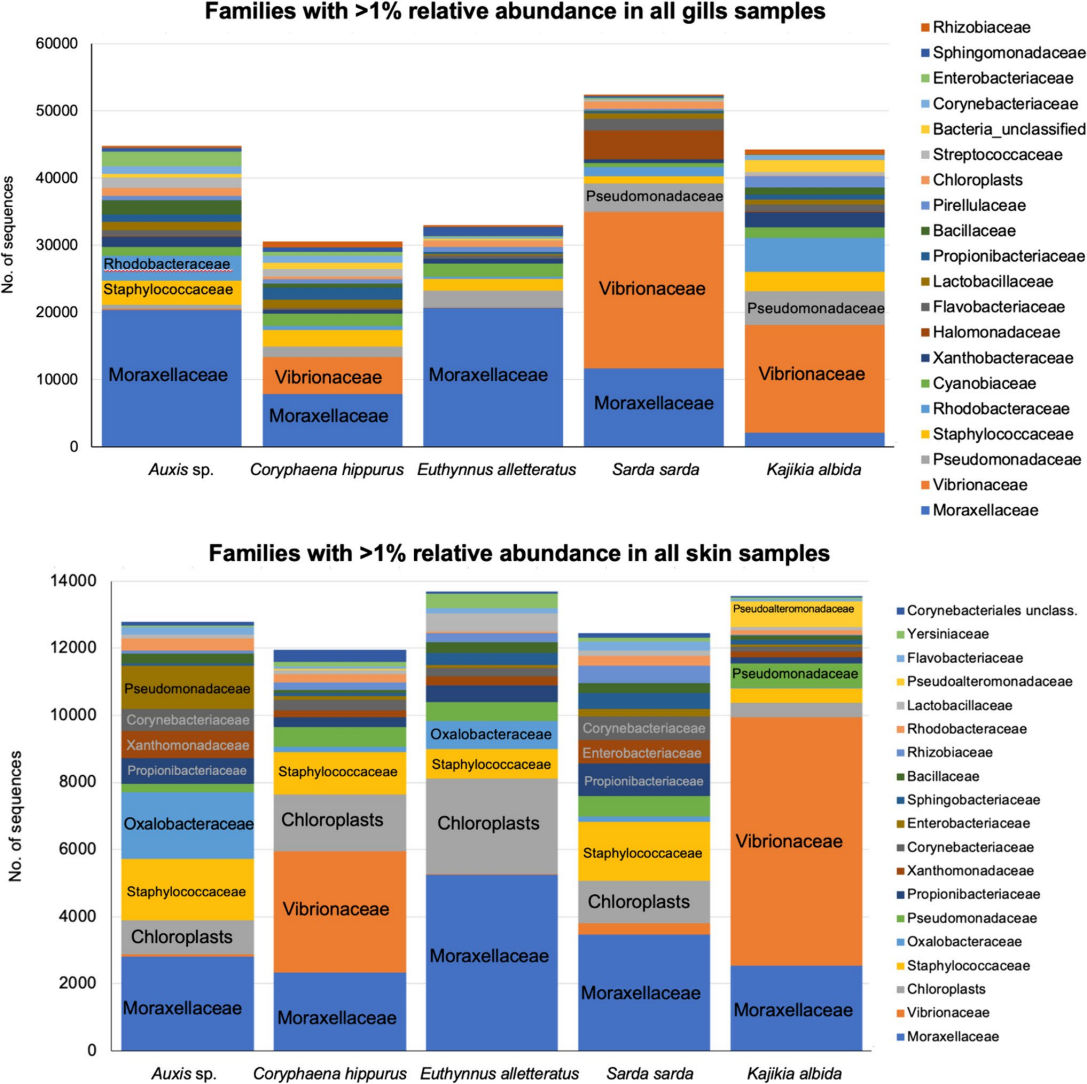
Abundance of the bacterial family-level operational taxonomic units with > 1% relative abundance in all gills (top) and skin (bottom) samples of five pelagic fishes from the Atlantic Ocean

OTUs from the Pseudomonadota family Moraxellaceae dominated both in the gills and skin of most fishes, with the exception of *S. sarda* and *K. albida* gills and *C. hippurus* and *K. albida* skin which were dominated by the Pseudomonadota family Vibrionaceae (Fig. 3). Other abundant families in the gills were the Staphylococcaceae and (*Auxis* sp.) and Pseudomonadaceae (*S. sarda* and *K. albida*), while in the skin, several other families of the Actinomycetota, Bacillota, and Pseudomonadota phyla and chloroplasts were also abundant.

**Fig. 4:**
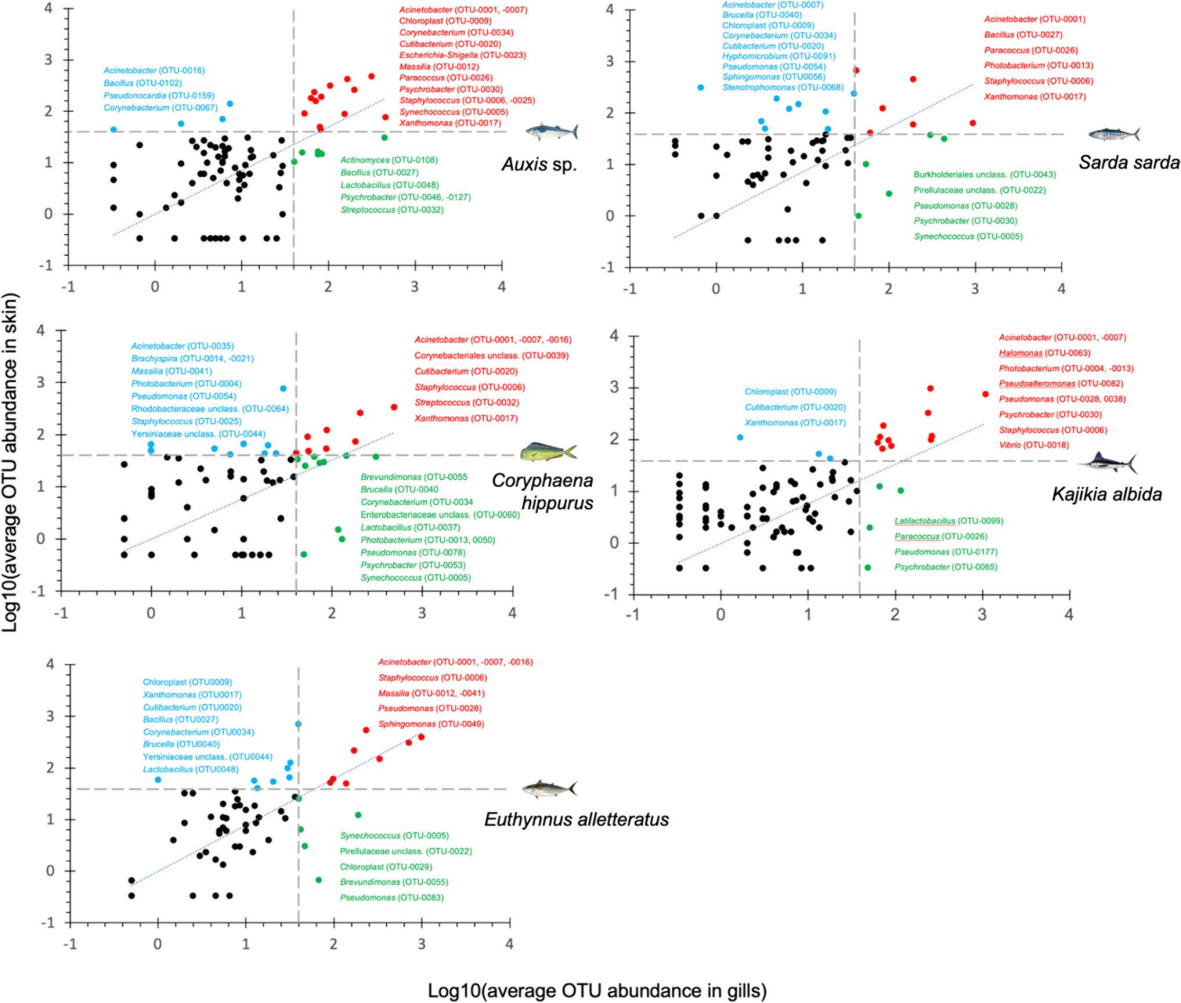
Operational taxonomic units with > 1% relative abundance (dashed grey line) in both gills and skin (red dots/letters), only in gills (green dots/letters) and only in skin (blue dots/letters) samples of five pelagic fishes from the Atlantic Ocean. Diagonal dotted line depicts OTUs with equal abundance in gills and skin samples, whereas those enriched in the skin or gills samples fall above or below the line, respectively

Each fish species harboured important OTUs, i.e. concomitant occurrence in both tissues and with ≥ 1% relative abundance (Fig. 4). *Acinetobacter*- and *Staphylococcus*-related OTUs were the two bacterial genera that fulfilled these two criteria in both tissues and all fishes, while OTUs affiliated with the *Xanthomonas* genus were found abundant in three of the five fishes (*Auxis* sp., *S. sarda*, *C. hippurus*). OTUs related to the *Corynebacterium*, *Cutibacterium*, *Massilia*, *Paracoccus* and *Psychrobacter* genera occurred as important genera only in various pairs of the investigated five fishes.

## Inferred metabolic functions of the gills and skin tissues

**Fig. 5:**
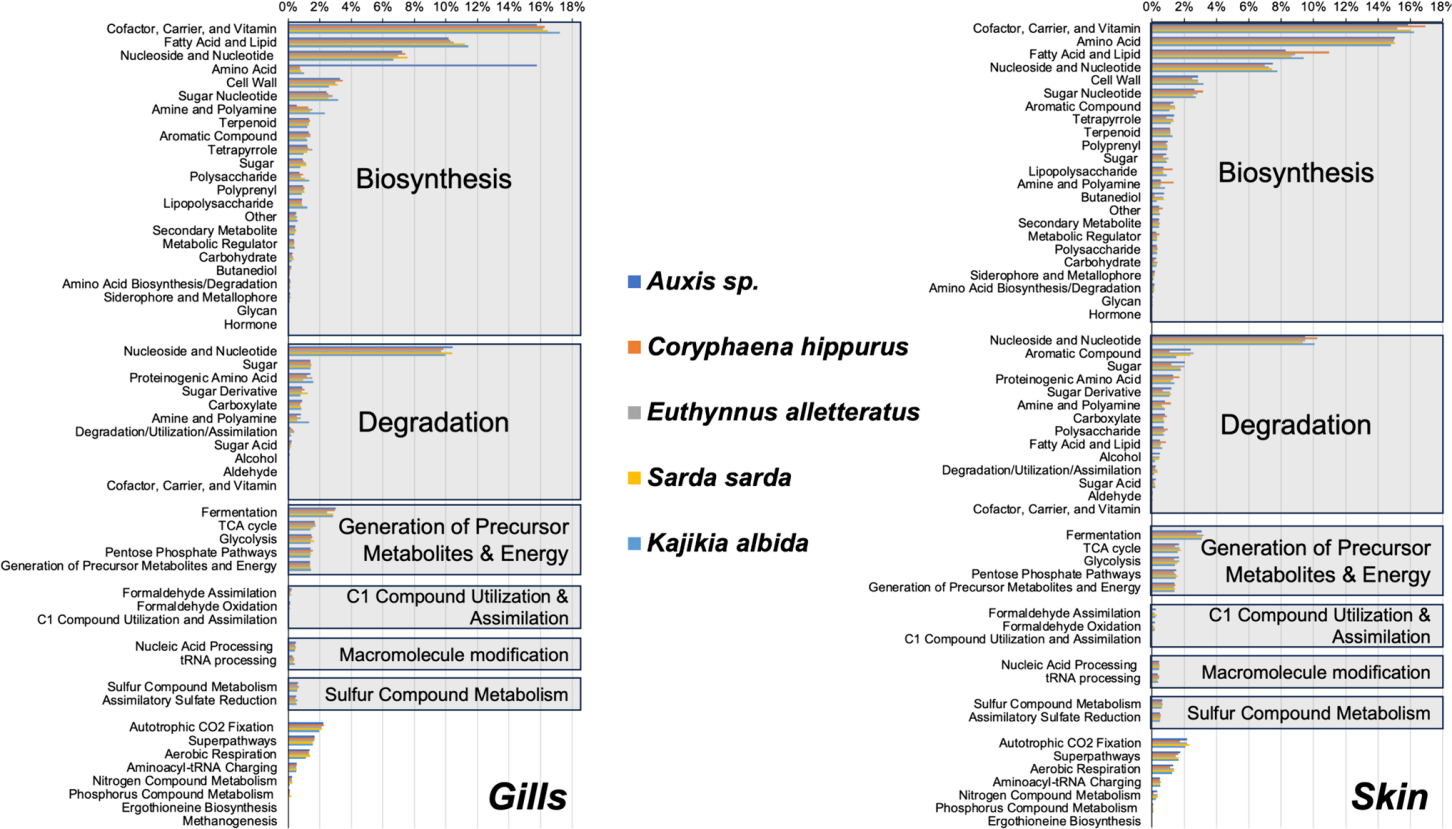
Inferred bacterial metabolic pathways between the gills and skin tissues in each of five pelagic fishes from the Atlantic Ocean

Between the two tissues, the overlap of the inferred bacterial metabolic pathways was ≥ 94% and was dominated by basic metabolism (Fig. 5). In the *Auxis* sp. gills, a considerably higher contribution, of amino acid (AA) biosynthesis, i.e. 15.8–22.5 times higher compared to the other four fishes, was observed. When broken down to specific AA biosynthesis in the gills, all five fishes have similar pathways, with most important being the biosynthesis of isoleucine (ile, 19 ± 1.3%), methionine (met, 16 ± 1.7%), lysine (lys, 12 ± 1.0%) and arginine (arg, 12 ± 0.6%) (Fig. S2).

## Discussion

Even to date, the fish microbiome literature is biased towards the intestinal tract (Legrand et al. 2020; Diwan et al. 2023), with much information missing about the fish gill and skin microbiome, despite the importance of these two tissues being the primary barrier to the fish’s internal environment. At the same time, these tissues are in immediate and constant contact with the surrounding water, and as such, they are affected by both environmental but also fish species-dependent factors (Larsen et al. 2013; Chiarello et al. 2018; Krotman et al. 2020; Minich et al. 2020a; Bruno et al. 2023). Even more under-studied are the gills and skin microbiomes of wild fish, as captivity seems to highly shape these microbiota (Uren Webster et al. 2020), and often they are related to health comprised populations of aquaculture farmed species. This leaves us with still very limited knowledge of the skin and gills microbiomes of wild fish.

In this paper, we aimed at depicting the important OTUs in the skin and gills of healthy specimens belonging to five wild fish species, shown as red dots in Fig. 4. We refer to these OTUs as likely core and obligate associates to the two tissues of their hosts (sensu (Hamady & Knight 2009; Shade & Handelsman 2012; Neu et al. 2021). It has been suggested that genera or higher taxonomic levels are appropriate for any effort to depict likely core microbiota (Neu et al. 2021), and for this, we present data on families and genera. Although we focus οn the core microbiota, the potential role of rare OTUs should not be overlooked as it has been shown that fish can change their abundance (Troussellier et al. 2017), and at least in the case of gut microbiota, the rare microbes have been recently shown to play an important role in immunological responses of the mammalian gut (Han et al. 2022). To date, no available data exist on the gills and skin microbiota/microbiome of the five fishes we investigated in this paper. The important genera we have depicted are likely to be established and actively growing members members of the microbial assemblages of the tissues which they were found, based on their high 16S rRNA gene copy numbers (Tab. S2) which is a proxy for fast growth (Roller et al. 2016).

The observed high overlap between gills and skin microbiota in the current study has also been previously reported in farmed marine fish, most likely due to the common exposure of the two tissues to the surrounding water; this is also supported by the occurrence of cyanobacterial/chloroplast-related OTUs (Legrand et al. 2018). A lower overlap (ca. 15%) between gills and skin microbiota reported by Minich et al. (2022) could be due to the high number of fish species included (68 in total) and also due to the fact that amplicon sequence variants were used instead of OTUs as in the present study.

*Acinetobacter* is the genus with the most likely role as a core microbiota member of the fishes investigated here. It has also been reported as an important genus in the gills and/or skin of closely related fish species. In wild *Thunnus albacares* and *T. obesus*, it has been found to dominate all investigated body parts (Zou et al. 2023) and has likewise been reported in the skin of farmed *Thunnus maccoyii* (Minich et al. 2020b). Its importance for the fish skin microbiota is also indirectly shown, as the genus showed decreased relative abundance in chronically stressed fish (Cámara-Ruiz et al. 2022). It has been found also in the skin core microbiota of farmed Atlantic salmon (Lorgen-Ritchie et al. 2022) and as dominant in *Sparus aurata* farmed larvae (Califano et al. 2017), which are more influenced by the surrounding sea water (Nikouli et al. 2019). Despite that *Acinetobacter* contains putative pathogenic species and strains, their presence is much more common in freshwater fish (Behera et al. 2017; Louvado et al. 2021; Bruno et al. 2023). Our three *Acinetobacter*-related OTUs are closely related (≥ 99% similarity) with potential freshwater fish gills and skin pathogens *Α. johnsonii*, *Α. lwoffii* (Kozińska et al. 2014), and *A. schindleri* (Zhao et al. 2022). However, all of the individuals investigated here did not have any visual signs of compromised health upon catch, suggesting that it is very likely that our *Acinetobacter*-related OTUs represent either commensal or beneficial bacteria for the gills and skin of the five marine fishes reported here. The occurrence of non-pathogenic microbes on the gills mucosal surface has already been reported by a number of workers (Diwan et al. 2023). Acinetobacter has been reported as a possible contamination in low-biomass samples (Salter et al. 2014; Weyrich et al. 2019) such as gills (François-Étienne et al. 2023), but our blank extractions did not give any amplifiable bacterial DNA, and, in addition, *Acinetobacter* was found in low and variable abundances in the gut samples of the same fishes (Varela, Papaspyrou, Kormas unpublished data).

The second most likely core and obligate associate genus with the two investigated tissues is *Staphylococcus*, and as for *Acinetobacter*, this is the first time it is reported as an important bacterial community component of the gills and skin of the five marine fishes of the present study. It has been found as one of the major genera found in the gills of three Mediterranean species (*Pagrus caeruleostictus*, *Scomber colias* and *Saurida lessepsianus*) (Itay et al. 2022) and the culturable bacterial fraction of farmed *Thunnus thynnus* gills and skin (Kapetanović et al. 2017). It has been proposed that when *Staphylococcus* is detected in healthy specimens and since pathogenicity is also influenced by abiotic and biotic factors, virulence of some bacterial taxa, such as *Staphylococcus*, is not obligatory and that potential pathogens do occur in healthy fish (Itay et al. 2022). Our results, with the high dominance of *Acinetobacter* and *Staphylococcus* in healthy specimens enhance this concept. The non-obligatory pathogenic feature of this genus has also been confirmed in farmed *Sparus aurata* individuals, where *Staphylococcus* fingerprints dominated in non-ulcered skin samples (Tapia-Paniagua et al. 2018). This genus has been reported among the important ones in the gut of other wild fish (Givens et al. 2015; Kormas et al. 2023). One of the two *Staphylococcus*-related OTUS is more like to belong to *S. epidermidis* (OTU0006 with ≥ 99.5% sequence similarity), while the other (OTU0026) had a much lower similarity (94%) with this species. *Staphylococcus* is a copiotroph, and so the mucus-rich fish skin could provide the right habitat for this genus to thrive (Larsen et al. 2013).

Although we cannot securely predict the exact functions of these bacterial communities, our results based on the inferred bacterial metabolic pathways showed that there is a high degree of functional redundancy in these communities and that there are little differences between the gills and skin. The dominant metabolisms are housekeeping functions, while some beneficial to the host include biosynthesis and degradation pathways of nucleic acids, proteins/amino acids and carbohydrates. The high functional overlap could reflect the seawater exposure of both tissues, as was the case in taxonomical diversity.

All five fishes showed similar patterns of bacterial metabolic pathways with the exception of *Auxis* sp. gills that had increased total AA biosynthesis. Enrichment of AA metabolism in fish skin has been related to metabolic pathways involved in the activation of immune responses (Guivier et al. 2020) and, thus, could be of relevance to defence mechanisms against pathogens colonizing the skin. The highest relative abundances of AA biosynthesis were observed for isoleucine, methionine, lysine and arginine. These AA are among the essential AA for several animals including fish, and, at least for the gut habitat, they are known to be important to the host’s energy acquisition via their fermentation to short chain fatty acids and/or acting as signalling molecules between the microbes and their host (McCann & Rawls 2023). Such an extra energy source in these large-bodied and fast-swimming fish could be of benefit, but whether these microbial traits from the gills and skin can actually benefit the five fishes of the current study remains to be investigated.

## Supplementary Information

Below is the link to the electronic supplementary material.

Supplementary file1 (PDF 457 KB)

## Data Availability

The raw DNA sequences from this study have been submitted to the Sequence Read Archive (https://www.ncbi.nlm.nih.gov/sra/) in the BioProject PRJNA1068742 (BioSample SAMN39602282). Reviewer link is https://dataview.ncbi.nlm.nih.gov/object/PRJNA1068742?reviewer=vs40eq6md08lfn4vv57vcaso1p.
