# Supplementary material for: The gills and skin microbiota of five pelagic fish species from the Atlantic Ocean
Source: Int Microbiol. 2024 May 13;28(1):95–105. doi: 10.1007/s10123-024-00524-8 (PMC11775069; doi:10.1007/s10123-024-00524-8)
Supplement: Supplementary file 1 — Supplementary file1 (PDF 457 KB) [file 10123_2024_524_MOESM1_ESM.pdf]

The gills and skin microbiota of five pelagic fish species from the Atlantic Ocean

José Luis Varela[1#], Eleni Nikouli[2#], Antonio Medina[1], Sokratis Papaspyrou[1], Konstantinos Kormas[2,3 *]

[1] Department of Biology, University of Cádiz, Puerto Real, 11510, Cádiz, Spain

[2] Department of Ichthyology and Aquatic Environment, School of Agricultural Sciences, 384 46 Volos, Greece

[3] Agricultural Development Institute, University Research and Innovation Centre "IASON", Argonafton & Filellinon, 382 21 Volos, Greece

[#] equal contribution

[*] Corresponding author: kkormas@uth.gr

SUPPLEMENTARY MATERIAL

Table S1. Morphometric data on the sampled fishes.

Species	Specimen	Length (cm)*	Total weight (g)	Sampling date
Auxis sp.	*1*	35.9	671.5	September 2019
Auxis sp.	*2*	29.6	346.5	September 2019
Auxis sp.	*3*	40.1	1093	September 2019
Coryphaena hippurus	*1*	64.8	2620.5	August 2019
Coryphaena hippurus	*2*	120.5	22000	August 2019
Euthynnus alletteratus	*1*	51.6	1849	August 2019
Euthynnus alletteratus	*2*	49.5	1790	August 2019
Euthynnus alletteratus	*3*	52.5	2227.5	September 2019
Sarda sarda	*1*	37.7	635.5	June 2019
Sarda sarda	*2*	41.6	900.5	June 2019
Sarda sarda	*3*	39.5	1791.5	June 2019
Kajikia albida	*1*	194	40400	September 2019
Kajikia albida	*2*	181	33000	September 2019
Kajikia albida	*3*	163.5	24200	September 2019

* Straight fork length, except for *Kajikia albida* which was lower-jaw fork length.

Table S2. Number of 16S rRNA gene copy numbers based on the rrnDB database*, of the important bacterial genera found in the gills and skin tissues in each of five pelagic fishes from the Atlantic Ocean (accessed on 07 Dec. 2023).

Genus	Average ± standard deviation of the 16S rRNA gene copy number	No. of genomes
Acinetobacter	6.2 ± 0.5	652
Corynebacterium	4.3 ± 0.8	351
Cutibacterium	3.0 ± 0.5	38
Propionibacterium	2.0 + 0.0	20
Massilia	6.6 ± 1.7	23
Paracococcus	2.7 ± 0.5	27
Psychrobacter	4.4 ± 0.9	18
Staphylococcus	5.7 ± 0.7	1161
Xanthomonas	2.0 ± 0.1	354

* Stoddard SF, Smith BJ, Hein R, Roller BRK, Schmidt TM (2014) rrnDB: improved tools for interpreting rRNA gene abundance in bacteria and archaea and a new foundation for future development. Nucleic Acids Research 43:D593-D598

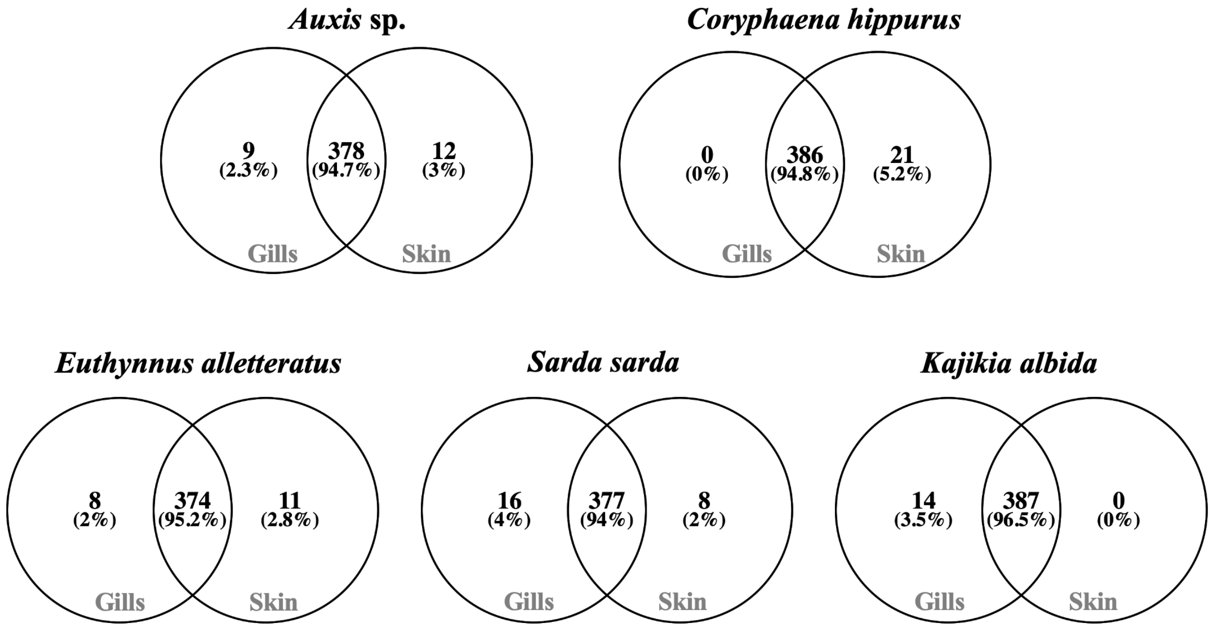

Figure S1. Overlap of the inferred bacterial metabolic pathways between the gills and skin tissues in each of five pelagic fishes from the Atlantic Ocean.

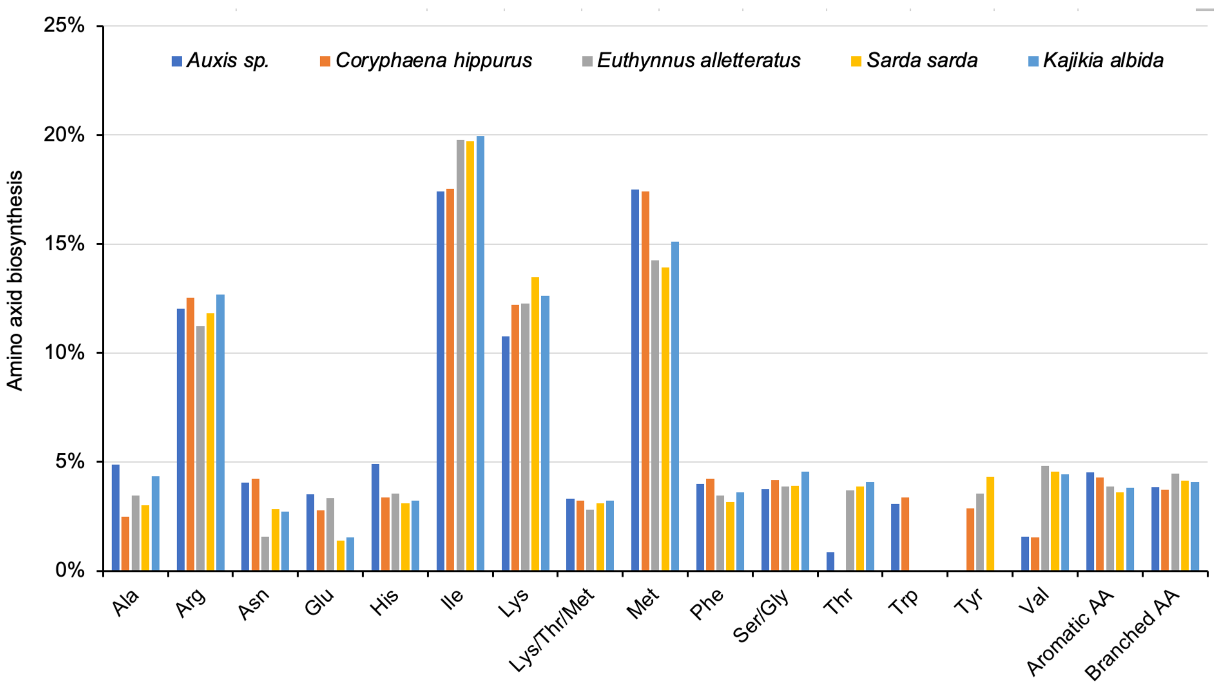

Figure S2. Relative contribution of the bacterial amino acid biosynthesis in the gills of five pelagic fishes from the Atlantic Ocean.
